# Effects of gait modifications on tissue‐level knee mechanics in individuals with medial tibiofemoral osteoarthritis: A proof‐of‐concept study towards personalized interventions

**DOI:** 10.1002/jor.25686

**Published:** 2023-09-10

**Authors:** Amir Esrafilian, Kimmo S. Halonen, Christine. M. Dzialo, Marco Mannisi, Mika E. Mononen, Petri Tanska, Jim Woodburn, Rami K. Korhonen, Michael S. Andersen

**Affiliations:** ^1^ Department of Technical Physics University of Eastern Finland Kuopio Finland; ^2^ Central hospital of Päijät‐Häme Lahti Finland; ^3^ Department of Materials and Production Aalborg University Aalborg Denmark; ^4^ Medere Roma Italy; ^5^ Griffith Centre of Biomedical and Rehabilitation Engineering, Menzies Health Institute Queensland Griffith University Gold Coast QLD Australia; ^6^ Center for Mathematical Modeling of Knee Osteoarthritis (MathKOA), Department of Materials and Production Aalborg University Aalborg Denmark

**Keywords:** finite element modeling, gait modification, knee osteoarthritis, lateral wedge insole, musculoskeletal modeling

## Abstract

Gait modification is a common nonsurgical approach to alter the mediolateral distribution of knee contact forces, intending to decelerate or postpone the progression of mechanically induced knee osteoarthritis (KOA). Nevertheless, the success rate of these approaches is controversial, with no studies conducted to assess alterations in tissue‐level knee mechanics governing cartilage degradation response in KOA patients undertaking gait modifications. Thus, here we investigated the effect of different conventional gait conditions and modifications on tissue‐level knee mechanics previously suggested as indicators of collagen network damage, cell death, and loss of proteoglycans in knee cartilage. Five participants with medial KOA were recruited and musculoskeletal finite element analyses were conducted to estimate subject‐specific tissue mechanics of knee cartilages during two gait conditions (i.e., barefoot and shod) and six gait modifications (i.e., 0°, 5°, and 10° lateral wedge insoles, toe‐in, toe‐out, and wide stance). Based on our results, the optimal gait modification varied across the participants. Overall, toe‐in, toe‐out, and wide stance showed the greatest reduction in tissue mechanics within medial tibial and femoral cartilages. Gait modifications could effectually alter maximum principal stress (~20 ± 7%) and shear strain (~9 ± 4%) within the medial tibial cartilage. Nevertheless, lateral wedge insoles did not reduce joint‐ and tissue‐level mechanics considerably. Significance: This proof‐of‐concept study emphasizes the importance of the personalized design of gait modifications to account for biomechanical risk factors associated with cartilage degradation.

## 1 INTRODUCTION

Knee osteoarthritis (KOA) is a degenerative joint disease reported as a leading cause of disability across the globe,^1^ affecting roughly one out of five adults over the age of 45 years.^2^ Joint stiffness, chronic pain, and functional disability caused by KOA profoundly affect the quality of life, influencing both physical and psychological health conditions.^3^ KOA is characterized by an irreversible deterioration of knee cartilages and it is often associated with the abnormal remodeling of the underlying bone.^4^ As there is no cure for the disease, the current treatments are targeted towards symptom control, starting with nonsurgical interventions, until the degree of KOA severity imposes the necessity of surgical intervention such as joint replacement.

Excessive mechanical loading of the knee has been strongly associated with the progression of KOA.^5^, ^6^ Accordingly, nonsurgical interventions, such as gait modifications, have been introduced to target KOA biomechanical risk factors. These interventions aim to alter lower extremity kinematics and kinetics to favorably reduce or redistribute knee joint contact forces (JCFs).^7^, ^8^, ^9^, ^10^, ^11^ Nevertheless, direct measurement of knee JCF is currently limited to subjects with instrumented knee implants.^12^ Due to this limitation, experimentally measured knee abduction/adduction moment is widely used as a surrogate measure for knee JCF to assess the effectiveness of different gait modifications.^7^, ^9^, ^10^, ^11^ However, it has been shown that changes in knee abduction/adduction moment and JCF might be poorly correlated to alterations within the local tissue‐level joint mechanics, such as knee cartilage stress and strain,^9^, ^13^ which are thought to govern tissue remodeling and degradation response.^5^, ^14^, ^15^ As a result, outcomes of gait modification treatments are controversial and may depend on the practitioners’ expertise.^8^, ^10^, ^16^, ^17^, ^18^

Due to the burden of experimental methods to assess detailed knee mechanics, physics‐based simulations have become a tool of choice to estimate joint‐ and tissue‐level knee mechanical responses.^8^, ^9^, ^11^, ^13^, ^19^, ^20^ Namely, musculoskeletal (MS) modeling has been widely used to assess alterations in knee kinematics and kinetics in different gait modifications such as toe‐in, toe‐out, and lateral wedge insoles.^8^, ^10^, ^19^ However, these studies have used MS models with rigid‐body knee joints incapable of accounting for subject‐specific knee articulating surfaces (e.g., cartilage defects) and omitted the interaction of ligaments and menisci with estimated knee kinematics and kinetics. Furthermore, MS models cannot estimate tissue‐level joint mechanics, such as stress and strain within the knee cartilage. Thorough knowledge of tissue‐level knee mechanics is essential to predict the degradation response of knee cartilage (e.g., due to gait modifications), such as collagen network damage, cell death, and loss of proteoglycans, for which finite element (FE) analysis has demonstrated a great potential.^5^, ^13^, ^14^, ^15^ Nevertheless, to the best of our knowledge, there are no studies assessing the effect of gait modifications on tissue‐level joint mechanics of individuals with KOA.

In this study, we aimed to (1) estimate tissue‐level knee mechanics of individuals with KOA undertaking different gait modifications and (2) investigate whether the optimal gait modification (i.e., the one with the most reduction in knee mechanics associated with KOA progression) differs when using subject‐specific tissue‐level knee mechanics (i.e., using FE analysis) compared with joint‐level knee mechanics (i.e., using MS analysis) or the conventional recommendations reported in the literature. To this end, we utilized our previously developed multiscale MS‐FE analysis workflow, accounting for subject‐specific kinematics, kinetics, and articulating surfaces of the knee.^13^, ^21^ We hypothesized that the optimum gait modification, which minimizes knee mechanics, might differ when using tissue‐level than joint‐level knee mechanics.

## 2 METHODS

### 2.1 Participants and data collection

Five participants with previously diagnosed medial tibiofemoral osteoarthritis^22^ were recruited for this study (Table 1). The study procedures, as well as the use of the collected data, were approved by the NHS Greater Glasgow and Clyde ethical committee (permission number 15‐WS‐0287 183203). Written informed consent was obtained from participants.

**Table 1:** Participants’ characteristics.

Patient number	Sex	Age (years)	Mass (kg)	Height (m)	Test leg	K/L grade^a^	
						Medial	Lateral
1	F	64	74	1.56	Right	4	2
2	M	60	112	1.84	Left	4	3
3	F	56	90	1.63	Right	4	2
4	M	74	89	1.66	Right	4	2
5	M	58	71.2	1.68	Left	4	2

Abbreviations: F, female; M, male.

^a^ Kellgren and Lawrence grade.

**Figure 1:**
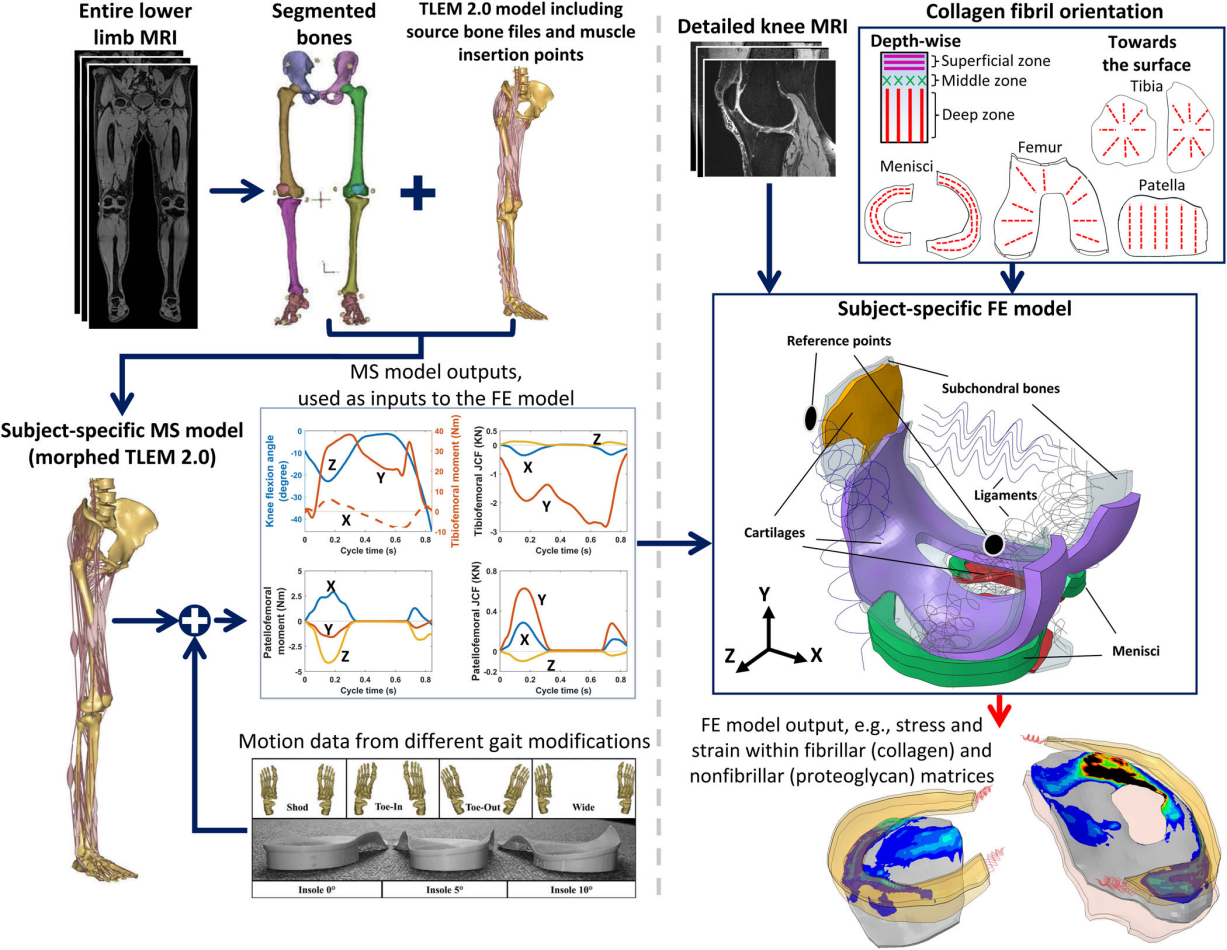
Workflow of the study (graphic is adapted from Dzialo et al.^8^).

The motion data were collected in the Human Performance Lab of Glasgow Caledonian University, consisting of three‐dimensional (3D) marker trajectories (Qualisys Opus camera system sampling at 120 Hz, Qualisys AB) and ground reaction forces (Kistler force plates sampling at 2000 Hz, Kistler Group). We studied a total of eight different gait conditions and modifications (Figure 1), consisting of normal barefoot walking (“barefoot”), normal shod walking (“shod”), walking with subject‐specifically made^23^ 0°, 5°, and 10° lateral wedge insoles (“insole‐0,” “insole‐5,” and “insole‐10”), walking with toes slightly turned inwards (“toe‐in”), walking with toes slightly turned outwards (“toe‐out”), and walking with a wider stance (“wide”). Gait modifications were bilateral and toe‐in, toe‐out, and wide‐stance were performed relative to participants’ normal walking line‐of‐progression. Participants walked at their self‐selected speed with standardized shoes.^24^ The walkway was long enough to reach stable gait speed. Five trials were recorded for each gait style.

In addition to the motion data, two sets of magnetic resonance images (MRIs) were taken from each participant (Figure 1), consisting of (1) MRI of the entire lower extremity and (2) detailed knee MRI from participants’ selected knee (Table 1) using a 3T Siemens Prisma scanner. For the lower extremity MRI (i.e., used to create subject‐specific MS models), a Peripheral Angio 36 coil was used in combination with a T1W‐Vibe‐Dixon sequence taken in the transverse plane (in‐plane resolution 1.4 mm, slice thickness 1.4 mm).^8^ The detailed knee MRI (i.e., used to create subject‐specific FE models) was acquired using a quad knee coil and sagittal 3D‐DESS‐WE sequence (in‐plane resolution 0.6 mm, slice thickness = 0.7 mm).

### 2.2 MS analysis

The MS modeling of the current study are explained in greater detail in our previous study,^8^, ^25^ and hence, here we provide a brief explanation.

The MS analyses were performed in AnyBody Modeling System (v 7.1, AnyBody Technology).^26^ Subject‐specific MS models were built based on the generic human body model from AnyBody Managed Model Repository (AMMR, v 1.6), which were updated to utilize Twente Lower Extremity Model (TLEM v 2.0).^27^ Using lower extremity MRI of participants, the pelvis, femur, tibia, patella, talus, and foot bones were first manually segmented in MIMICS (v 19, Materialise). These segmented bones were used to morph the cadaver‐based stereolithography files from the TLEM v 2.0^27^ to the subject‐specific shapes using a Radial‐basis Function approach (Figure 1). Through the morphing, muscle insertion points and muscle wrapping surfaces were made subject‐specific.^8^ Additionally, subject‐specific hip centers, knee axes, and ankle axes were obtained through surface fits. In the MS models, the hip joint was modeled with a ball‐and‐socket (3 DoF) joint, and tibiofemoral, patellofemoral, ankle, and subtalar joints were modeled as hinge‐type (1 DoF) joints. Hill‐type muscle‐tendon units were used and muscles’ isometric strength were obtained based on body mass index, segments’ mass, and segments’ length.^28^ For each walking trial, the muscle forces ($f_{i}^{m})$ were estimated by minimizing the following cost function^8^, ^25^:

(1.a) $$\mathrm{Cost}\mathrm{function}=\sum_{i=1}^{M}v_{i}{\left(\frac{f_{i}^{m}}{N_{i}}\right)}^{3}$$

subjected to:

(1.b) Cf=d

(1.c) $$0\leq f_{i}\leq N_{i},\;i=1,2,\ldots ,\;M,$$

where *v*_i_ is the muscle volume normalization factor of the *i*th muscle,^25^, ^29^ $f_{i}^{m}$ is the force generated by the *i*th muscle, *N*_i_ is the instantaneous maximum isometric force of the *i*th muscle, and *M* is the total number of muscles in the MS model. Eq. 1.b represents equations of motion, in which **C** is a coefficient matrix for all the unknown forces (**f**) in the MS model, **f** is the vector of unknown forces consisting of muscle ($f_{i}^{m}$) and JCFs, and **d** is a vector containing all inertial, Coriolis, and external forces applied to the MS model.

Of note, research studies have shown that the cost function in Eq. 1.a, which minimizes the sum of cubed muscle forces, provides a more accurate estimation of knee JCF and muscle activations than commonly used criterion of the sum of squared muscle forces.^25^, ^30^ Moreover, in the TLEM,^27^ muscles with a wide insertion area are subdivided into multiple branches. However, such a subdivision of muscles can affect the muscle and joint reaction force estimates.^31^ Consequently, previous studies^25^, ^29^, ^32^ have defined a normalization factor (*v*_i_ in Eq. 1.a) based on the muscle volume, which accounts for a proper subdivision of the force among split and nonsplit muscles.

### 2.3 FE analysis

#### 2.3.1 Creating subject‐specific FE models

Detailed knee MRI of the participants were used to create subject‐specific FE models (Figure 1). The distal femur and proximal tibia from the detailed knee MRI were used to register the lower limb and detailed knee scans. Knee joint cartilages (consisting of femoral, tibial, and patellar cartilages), the underlying subchondral bones, and menisci were manually segmented in MIMICS (v 19, Materialise), and then the 3D geometries were meshed in HyperMesh (v 2019, Altair) using C3D8P elements. We were not interested in the subchondral bone mechanics; however, they were modeled to bear the load and provide contact in the regions where the cartilage was utterly worn out. Thus, only a thin layer of each subchondral bone (with several millimeters of thickness) was modeled to minimize the computational costs. Menisci horn attachment and knee ligament insertion points were also extracted from participants’ MRI. Anterior and posterior cruciate ligaments (ACL and PCL), medial and lateral collateral ligaments (MCL and LCL), medial and lateral patellofemoral ligaments (MPFL and LPFL), and patellar ligament (connecting patella to the tibia) were included in the FE models. Ligament properties, consisting of prestrain and stiffness, were based on our previous studies,^21^, ^33^, ^34^, ^35^, ^36^, ^37^, ^38^ in which we observed knee secondary kinematics and center of pressure (CoP) comparable to in vivo and in vitro data.

Subchondral bones were modeled as linear elastic (Young's modulus = 15 GPa, Poisson's ratio = 0.3^39^). Knee cartilages were modeled using a fibril‐reinforced poroviscoelastic (FRPVE) material model and menisci were modeled using a fibril‐reinforced poroelastic material model^21^ (Figure 1). These biphasic FRP(V)E material models have been developed to account for the interaction of fibrillar (i.e., collagen network) and nonfibrillar (i.e., proteoglycan) matrices, as well as the interstitial fluid flow within the knee cartilages and menisci.^13^, ^21^, ^35^, ^40^, ^41^ The FRP(V)E material parameters of healthy tissues were obtained from the literature, as no studies reported material parameters for osteoarthritic human tissue, that is, verified at joint level analysis. ACL, PCL, MCL, and LCL were modeled using nonlinear spring bundles,^21^, ^35^, ^42^ whereas MPFL, LPFL, patellar tendon, and menisci horn attachments were modeled using linear spring bundles.^21^, ^35^, ^43^, ^44^, ^45^ More details on the material models and parameters can be found in Supporting Information: Section 1.1.

#### 2.3.2 Boundary conditions, loading, and FE analysis

The FE models had a 6‐DoF tibiofemoral and a 6‐DoF patellofemoral joints, and contact interactions were defined to include all the possible contacts, that is, cartilages, menisci, and subchondral bones. We exploited a kinematics‐kinetics‐driven MS‐FE modeling approach, developed and verified in our previous studies,^13^, ^21^, ^34^, ^35^ to provide the FE models with inputs. Of note, the modeling approach has shown great potential for estimating knee JCF^25^, ^33^, ^35^ and contact pressure,^34^ and predicting locations susceptible to osteoarthritis.^37^, ^38^

First, two reference points (i.e., femoral and patellar) were defined within the FE models according to the origin of the femoral and patellar coordinate systems of the associated MS model (Figure 1). Then, femoral and patellar cartilages and subchondral bones were coupled to the reference points, correspondingly. The FE models’ inputs were applied to the femoral and patellar reference points, whereas all the nodes on the bottom of the tibia (i.e., either tibial cartilage or tibial subchondral bone) were fixed to the ground (Figure 1).

Inputs to the FE models (Figure 1) consisted of (1) knee flexion angle, (2) the net forces and moments on femur coming from the gravitational, inertial, muscle, and hip joint reaction forces, and (3) the net forces and moments applied on patella coming from the gravity, inertia, and the quadriceps muscles. The FE analyses were performed in Abaqus (v 6.20, Dassault Systèmes) using soil consolidation analysis and the whole stance phase of each gait trial was analyzed. FE analysis took ~15 h for each trial on an Intel Xeon 6248 CPU (single‐core analysis). More details on the boundary conditions can be found in Supporting Information: Section 1.2.

### 2.4 Postprocessing of the results

The FE analysis could be successfully completed for 141 out of 200 study trials (≥2 trials per subject per gait style, except for the toe‐in modification of participant 1, toe‐out modification of participant 4, and wide modification of participant 5). FE analysis of the rest of the trials (59 trials) did not converge due to mesh distortion caused by the excessively worn cartilages during the analysis. Here we made overall comparisons across the participants and gait conditions and modifications, using the mean profile of the successfully analyzed trials. Also, we compared different gait modifications to shod walking, assuming that it is the most conventional footwear in daily activities.

We investigated secondary knee kinematics, tibiofemoral JCF, tibial cartilage CoP, and maximum and impulse of mechanical responses of knee cartilages, including maximum principal stress, maximum shear strain, and collagen fibril strain. Here, secondary knee kinematics were estimated within FE analysis and refer to the tibiofemoral and patellofemoral DoF, excluding knee flexion (primary knee kinematics estimated using inverse kinematics). Also, the force passing through the subchondral bone refers to the force transmitted only through the contact regions between the tibial subchondral bone and either femoral cartilage, femoral subchondral bone, or menisci (i.e., excluding the JCF passing through the tibial cartilage). However, the tibiofemoral JCF refers to the sum of the forces passing through the tibial cartilage and its underlying subchondral bone.

To calculate the maximum and impulse of tissue mechanics, first, the elements of each compartment (e.g., medial tibial cartilage) that were in contact with any other surfaces (such as femoral cartilage, bones, and menisci) were detected separately at each time point. From these elements, the element with the maximum tissue mechanical response and its neighboring elements (elements sharing ≥ 1 node) were selected at each time point of the cycle. This was done separately for maximum principal stress, maximum shear strain, and collagen fibril strain. Next, the average of the tissue mechanical response was calculated from these selected (neighboring) elements, resulting in curves of the instantaneous maximum tissue mechanical responses over the contact area. From these, the maximum values (i.e., the peak of the trial) and the area under the curves (i.e., the time integral called the impulse) were calculated. The maximum values are discussed in the manuscript, while impulses of tissue mechanical responses are presented in the Supporting Information for interested readers.

## 3 RESULTS

**Figure 2:**
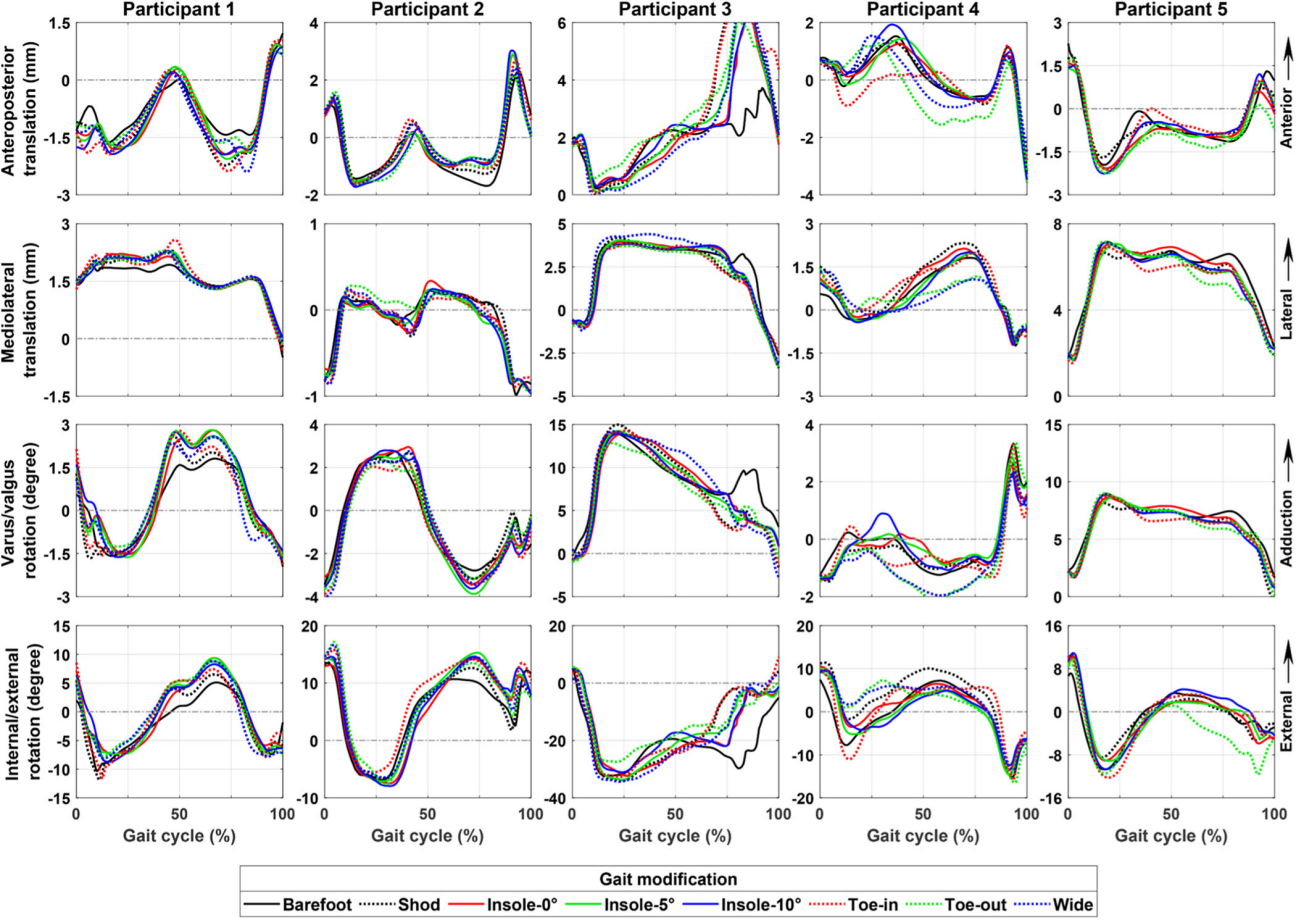
Secondary knee kinematics (i.e., femur relative to tibia) of study participants during walking with different gait modifications estimated by the FE models of the study. Plots show the average profile of each subject's gait modification.

**Figure 3:**
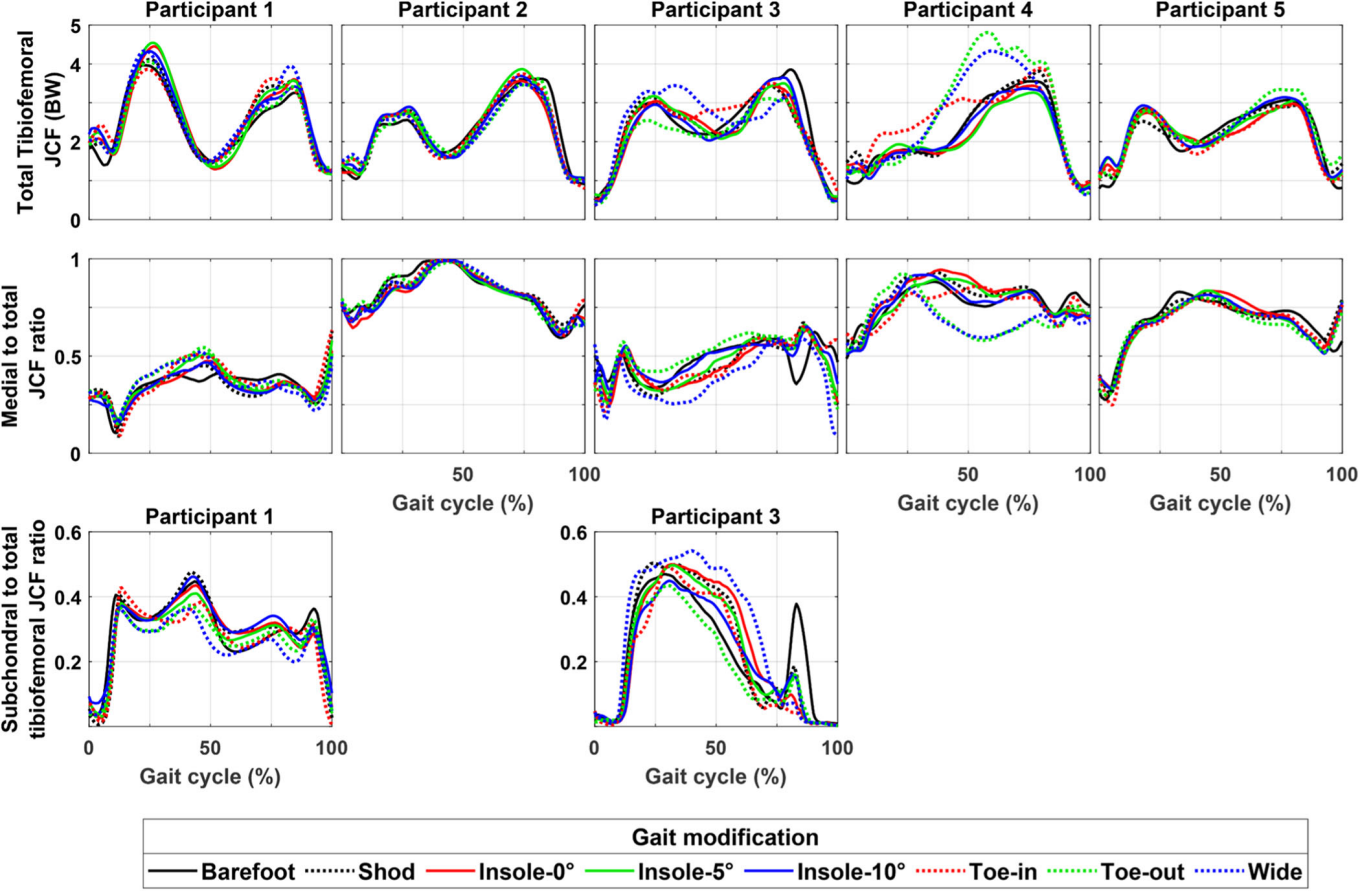
The tibiofemoral joint contact force (JCF) of the participants during walking with different gait modifications estimated by the finite element (FE) models of the study. Plots show the average profile of each subject's gait modification. Top row: total JCF. Middle row: the ratio of the medial JCF to the total JCF. Bottom row: the ratio of the JCF passing through the tibial subchondral bone to the total tibiofemoral JCF (patients with no plots mean zero JCF through the subchondral bone during the cycle). Note that the JCF passing through the tibial subchondral bone is the JCF passes through the contact area between the tibial subchondral bone and either of femur or menisci.

For two out of five participants, that is, Participants 2 and 5, the gait modifications had minor effects (near complete overlap) on the peak of the secondary knee kinematics (Figure 2) and total tibiofemoral JCF as well as medial to total tibiofemoral JCF (Figure 3). Across all the participants, the use of lateral wedge insoles caused only minor alterations in the estimated secondary knee kinematics, tibiofemoral JCF, and the CoP on the medial tibial cartilage compared with toe‐in, toe‐out, and wide gait modifications (Figures 2, 3, and Supporting Information: Figure S1).

**Figure 4:**
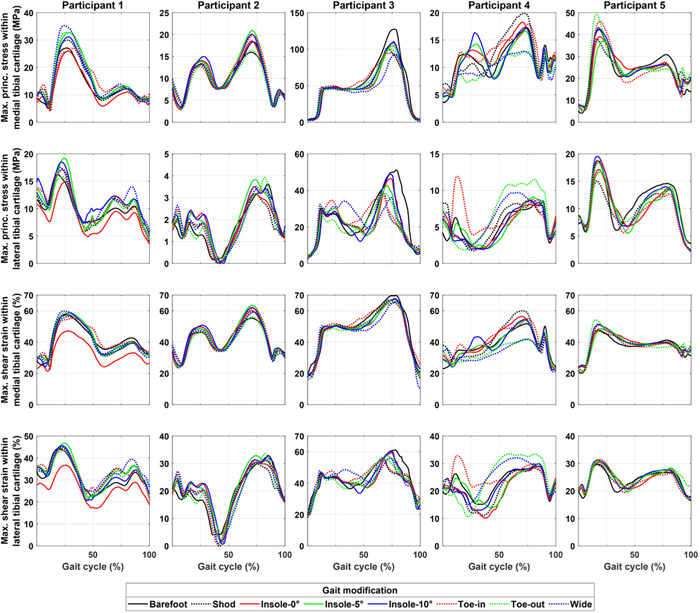
The peak of the maximum principal stress (previously suggested as the indicator of collagen network damage) and maximum shear strain (previously suggested as the indicator of proteoglycan loss and cell death) within the medial and lateral tibial cartilage of study participants during the gait cycles of different gait modifications. Plots show average profile of each gait modification.

**Figure 5:**
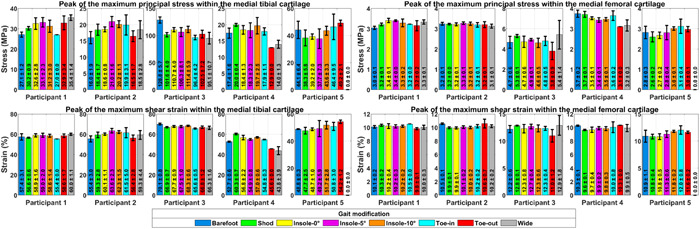
The peak of the maximum principal stress (top row) and maximum shear strain (bottom row) within the medial tibial cartilage (on the left) and medial femoral cartilage (on the right) of study participants. Error bars show SDs. The corresponding magnitudes (mean ± SD) are shown in the bars for ease of reading.

We observed measurable changes in the peak of the tissue‐level joint mechanics in all study participants across different gait modifications in both tibial (Figures 4 and 5) and femoral cartilages (Figure 5 and Supporting Information: Figure S2). Nevertheless, the gait modification that minimized tissue mechanics (e.g., the maximum principal stress, maximum shear strain, and collagen fibril strain) differed across the study participants and, in some cases, also across the region of interest (i.e., tibial or femoral cartilages). Regarding the lateral wedge insoles, our results did not show an overall decreasing (or increasing) trend in the peak of tissue mechanics in medial tibial and femoral cartilages when increasing the inclination angle of the insoles from 0 to 5 and 10 degrees (Figures 4, 5, and Supporting Information: Figures S1–S3).

**Figure 6:**
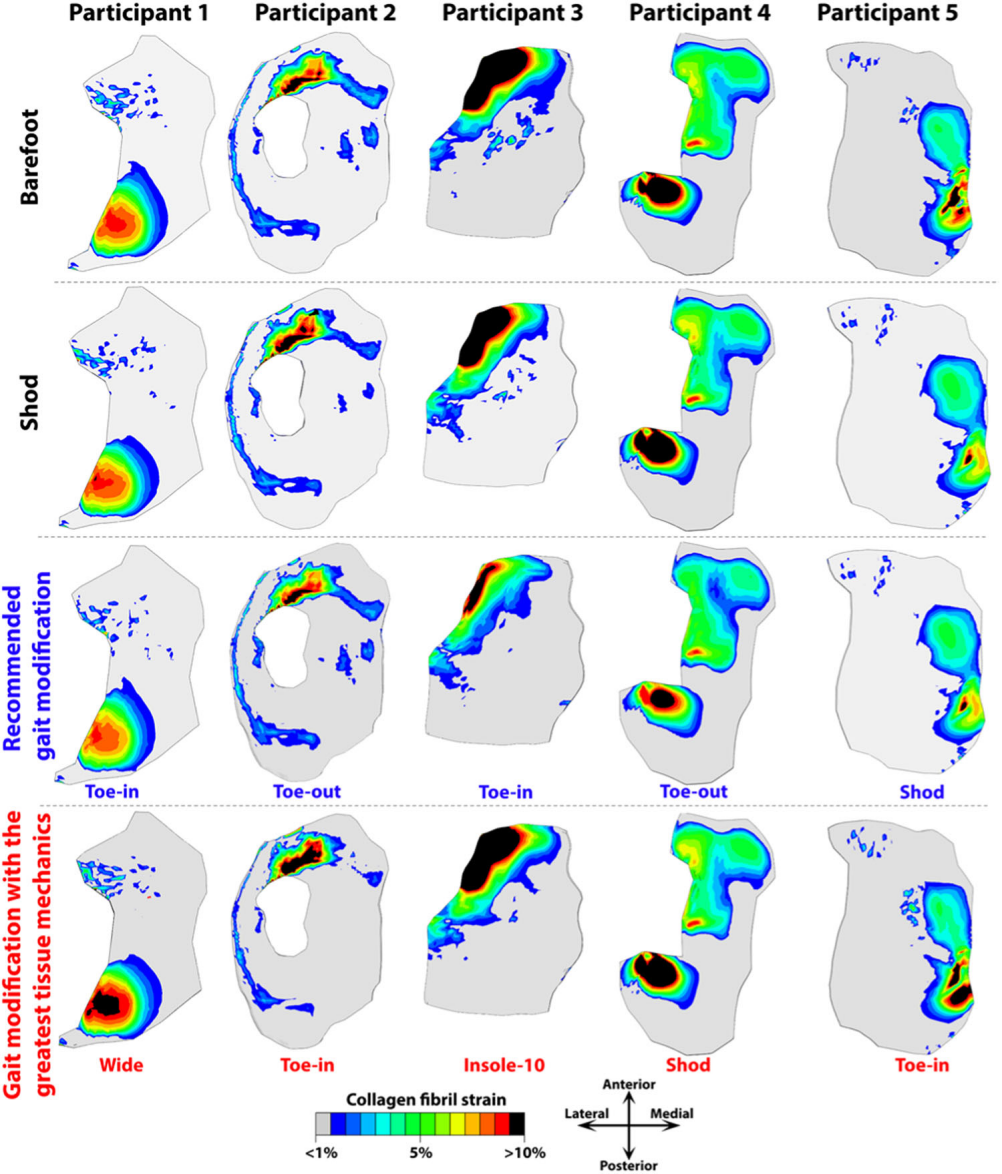
Contour plots of the collagen fibril strain within the medial tibial cartilage of study participants. Plots belong to the timepoints with maximum collagen fibril strains during barefoot walking (first row), shod walking (second row), as well as the gait modifications with the greatest reduction (third row) and increase (fourth row) in peak of tissue‐level mechanics within medial tibial and femoral cartilages.

In Participant 1, the peak of the tissue mechanical responses was the lowest during barefoot and toe‐in walking within the medial tibial cartilage, while wide stance modification was the one with greatest changes in tissue mechanics. That is, it increased the maximum principal stress by about +17% and the maximum shear strain by about +7% within medial tibial cartilage compared with shod walking (Figures 4, 5, 6). In Participant 1, walking with lateral wedge insoles increased tissue mechanics within the medial tibial cartilage (Figure 5 and Supporting Information: Figure S2). Changes in the medial femoral cartilage tissue mechanics across the modifications were marginal in participant 1 (Figure 5 and Supporting Information: Figure S2).

In Participant 2, toe‐out was the gait modification that produced the greatest reduction in tissue mechanics of medial tibial cartilage (Figures 4, 5, 6). Toe‐out also decreased tissue mechanics in medial femoral cartilage, but to a lesser degree than tibial cartilage (Figure 5 and Supporting Information: Figure S2). Compared with shod walking, toe‐out reduced the maximum principal stress by about −11% within the medial tibial cartilage (Figure 6). Nonetheless, in Participant 2, toe‐in and insole‐5 were the gait modifications with the greatest increase in the tissue mechanics in medial tibial and femoral cartilages, that is, about +14% in maximum principal stress and +7% in maximum shear strain within medial tibial cartilage, compared with shod walking (Figures 4, 5, 6 and Supporting Information: Figure S2).

In Participant 3, toe‐in and wide stance were the gait modifications, which produced the greatest reduction in medial tibial cartilage tissue mechanics, that is, about −6% in maximum principal stress of medial tibial cartilage compared with shod walking. Toe‐in also decreased tissue mechanics in medial femoral cartilage; however, toe‐out was the modification with the greatest reduction in medial femoral cartilage tissue mechanics, that is, about −28% in maximum principal stress and −12% in maximum shear strain compared with shod walking. In Participant 3, barefoot walking and insole‐10 produced the greatest increase in tissue mechanics within medial tibial cartilage, that is, up to +26% in maximum principal stress and +5% in maximum shear strain compared with shod walking (Figures 4, 5, 6 and Supporting Information: Figure S2).

In Participant 4, toe‐out and wide stance were the gait modifications, which produced the greatest reduction in both medial tibial and femoral cartilages tissue mechanics, that is, about −37% in maximum principal stress and −26% in maximum shear strain compared with shod walking (Figures 4, 5, 6 and Supporting Information: Figure S2). Nonetheless, shod walking produced the greatest tissue mechanics within the medial tibial and femoral cartilages, that is, about +15% in maximum principal stress and +14% in maximum shear strain of medial tibial cartilage compared with barefoot walking (Figures 4, 5, 6 and Supporting Information: Figure S2).

In Participant 5, shod walking produced the least tissue mechanics load within medial tibial and femoral cartilages, that is, about −13% in maximum principal stress and −2% in maximum shear strain of medial tibial cartilage, compared with barefoot walking (Figures 4, 5, 6 and Supporting Information: Figure S2). Toe‐in and toe‐out were modifications with the greatest increase in tissue mechanics within tibial and femoral cartilages, that is, about +28% in maximum principal stress and about +14% in maximum shear strain of medial tibial cartilage compared with shod walking (Figures 4, 5, 6 and Supporting Information: Figure S2).

## 4 DISCUSSION

In this study, using subject‐specific MS‐FE analysis, we investigated whether and how different gait modifications affected knee cartilage mechanics in individuals with medial tibiofemoral KOA. The MS models were generated using participants’ lower limb MRI, accounting for subject‐specific joints’ center, muscle insertion points, and muscle moment arms. Then the participants’ FE models were generated using manually segmented tibial, femoral, and patellar cartilages and subchondral bones, menisci, and knee ligaments. The FRPVE FE models enabled the estimation of tissue mechanics within fibrillar (i.e., collagen network) and nonfibrillar (i.e., proteoglycans) matrices of knee cartilages. Of note, excessive maximum principal stress and collagen fibril strain have been reported to indicate collagen network damage,^5^, ^6^ whereas excessive maximum shear strain is reported to indicate cell death and loss of proteoglycans^14^, ^46^ in knee cartilage.

Our results suggested that conventional approaches, that is, based on joint‐level mechanics, might fail in providing participants with the most appropriate gait modification to minimize the tissue mechanics and, potentially, postpone the onset and progression of KOA (Figure 6). More importantly, the use of subject‐specific tissue‐level mechanics (here obtained from FRPVE FE modeling) showed potential for differentiating the effect of gait modifications on the knee mechanics, which was not evident using joint‐level mechanics obtained from MS analysis (e.g., Participants 2 and 5 in Figure 3 compared with Figures 4, 5, 6). This was in line with our hypothesis and can be attributed to the inclusion of participant's joint geometries, the interaction of knee ligaments, and the use of the complex FRPVE material model in the FE models, compared with the rigid‐body MS analysis.^47^ It has been shown that tissue‐level knee mechanics can be substantially affected by alterations in knee articulating surfaces, for example, due to cartilage defect, which is common in participants with KOA.^14^, ^47^ Moreover, due to muscle weakness,^48^ individuals with KOA often have limited capability to coordinate their muscles, for example, to favorably control knee alignment, (un)load the knee, and reduce the load‐related pain.^49^ This limited capability may emphasize the importance of evidence‐based and subject‐specific design of gait modifications in individuals with KOA to avoid excessive tissue‐level loading^13^, ^19^ and reduce pain.^50^, ^51^

Nevertheless, there were also agreements between our study results and those reported in the literature, namely the controversial effectiveness of lateral wedge insoles. Lateral wedge insoles intend to shift the ground reaction force towards the outside of the foot and consequently reduce the knee adduction moment.^7^, ^50^ However, it is widely reported that the use of lateral wedge insoles provides small or almost no symptomatic or structural benefits for patients with KOA, compared with flat insoles.^16^, ^17^, ^52^, ^53^ Our results also showed a minimum (and in some cases, a detrimental) effect of the lateral wedge insoles on the knee kinematics (Figure 2), JCF (Figure 3), and tissue‐level knee mechanics (Figures 4, 5, 6 and Supporting Information: Figures S2 and S3). Hence, in line with the literature, our results indicated that using lateral wedge insoles is unlikely to decelerate the progression of mechanically induced KOA.

We also provided examples of the subject‐specific design of gait modification, that is, to reduce tissue‐level joint mechanics in the interest of decelerating or postponing KAO (Figure 6). Importantly, the participant's most‐appropriate gait modification (i.e., the one with the greatest reduction in knee cartilage mechanics) was different when using tissue‐level mechanics (such as stress and strain) compared with using joint‐level knee mechanics (such as knee abduction/adduction moment and JCF^8^). Overall, the differences originated from the stress concentration (e.g., at lesions or edges) and the nonlinear response of the FRPVE material model. Likewise, our previous study also showed that joint‐level mechanics (e.g., moments and JCF) are unlikely to represent tissue‐level mechanics (such as stress and strains)^13^ governing tissue degradation and remodeling response. For instance, in Participant 5, toe‐in was the gait modification with the greatest reduction in the medial tibiofemoral JCF (Figure 2, consistent with our previous study on the same participant^8^). In contrast, our FE analysis showed that toe‐in caused the greatest tissue‐level knee mechanics compared with other modifications (Figure 6), as CoP moved towards the posterior and lateral edge of the medial tibia (Supporting Information: Figure S1), causing stress concentration (Figure 6). To summarize, our subject‐specific analysis suggested the potential for enhancing the effectiveness of gait modifications, that is, planning for the reduction of tissue‐level joint mechanics to slow down or postpone KOA.

Our study also had limitations. First, we did not evaluate the effect of long‐term use of gait modifications, which may alter the outputs. Nevertheless, the utilized workflow has shown potential for analysis of follow‐up assessments.^14^, ^54^ Also, some gait modifications (i.e., toe‐in, toe‐out, and wide stance) were not standardized, as this would be difficult to achieve in practice. We pragmatically allowed these modifications to be determined by the participant based on comfort and feasibility to maintain the adaptation over a distance. The use of a 1 DoF knee joint within the MS analyses may be addressed as a study limitation. However, previous studies have shown that an MS model with a subject‐specific 1 DoF knee model could estimate knee JCF comparable with in vivo measurements^25^, ^55^ and also with the JCF estimated by an MS model with subject‐specific 12 DoF knee joint.^25^ Moreover, it has been reported that a 12 DoF knee FE model driven by a 1 DoF knee MS model could estimate secondary knee kinematics favorably comparable with the in vivo measurements, with those estimated by a 12 DoF MS‐FE model, and those estimated by a 12 DoF MS model.^33^, ^34^, ^56^

Static optimization has demonstrated the potential for detecting changes in knee JCF induced by gait modifications,^8^, ^57^ although muscle recruitment strategies may differ in patients with KOA^58^ than in normal individuals or those with knee implants (i.e., used in the study by Marra et al.^25^). Future studies should investigate methods to improve the included muscle recruitment criteria, for example, including co‐contractions explicitly in the MS analysis. One suggestion could be electromyography (EMG)‐assisted MS models.^33^, ^34^, ^35^, ^59^ However, EMG‐assisted approaches typically include simplifications of the underlying anatomy, frequently leave out the effects of deep muscles, and as such, may not show more accurate estimates of the in vivo joint forces when compared with instrumented implant data.^25^, ^60^ Nevertheless, here we were unable to explicitly verify estimated muscle activations or utilize EMG‐assisted MS‐FE pipelines due to the lack of EMG measurements in our data set. Also, muscle strengths within the MS models were set according to the literature,^8^, ^28^ since study participants were unable to perform maximum isometric strength measurements. Moreover, the material parameters, structure, and composition of knee cartilages, menisci, subchondral bones, and ligaments may vary across the individuals due to, for example, aging and tissue deterioration.^46^ However, the fact remains that there are no practical methods to fully extract subject‐specific material properties of knee load‐bearing tissue. Additionally, previous investigations have reported that measurement errors and uncertainties can substantially affect the calibration of material model parameters, for example, in ligaments.^61^ Hence, the material parameters of the FE models were adopted from the literature, including our previously verified MS‐FE models. Nonetheless, it has been reported that the use of softer or stiffer materials (i.e., representative of healthy or osteoarthritic cartilage) may change the magnitude of the estimated tissue mechanics, but the pattern and distribution of tissue mechanics remain comparable.^54^ Importantly, our investigations in the current study did not focus on the magnitudes of the estimated tissue mechanics, but we compared the relative tissue mechanics across the undertaken gait modifications. Moreover, gait modifications had a marginal effect on the knee CoP (Supporting Information: Figure S1) across the gait modifications. This potentially attenuates alterations in relative tissue mechanics (e.g., at maximum knee JCF) due to neglecting possible regional changes in material properties.

In conclusion, our proof‐of‐concept study suggested that the optimal gait modification of individuals may be different (and even opposed) when using tissue‐level mechanics, compared with using joint‐level knee mechanics such as knee abduction/adduction moment and JCF. We also demonstrated examples of using multiscale simulations to assist in choosing optimal gait modification producing minimum tissue mechanics, which may decelerate or postpone KOA progression. This offers great potential for clinicians’ decision‐making regarding the subject‐specific design of gait modifications. In the future, with a larger cohort and follow‐up data, we will expand and validate this proof‐of‐concept study by adapting our rapid (several minutes) MS‐FE modeling techniques^35^, ^62^ and cartilage degradation algorithms^5^, ^14^ to provide an automated in silico tool for subject‐specific design of nonsurgical corrective healthcare plans, such as gait modifications, gait retraining, and rehabilitation protocols for individuals with KOA.^13^

## AUTHOR CONTRIBUTIONS

**Amir Esrafilian**: Conceptualization; finite element modeling; interpretation of the data; writing—original draft. **Kimmo S. Halonen**: Assisting with finite element modeling; review and editing. **Christine. M. Dzialo**: Musculoskeletal modeling; review and editing. **Marco Mannisi**: Data collection; musculoskeletal modeling; review and editing. **Mika E. Mononen** and **Petri Tanska**: Interpretation of the data; review and editing. **Jim Woodburn**, **Rami K. Korhonen**, and **Michael S. Andersen**: Conception and design; musculoskeletal modeling; supervision; funding acquisition; review and editing.

## CONFLICT OF INTEREST STATEMENT

The authors declare no conflict of interest.

## Supporting information

Supporting Information.
