# Supplementary material for: Effects of gait modifications on tissue‐level knee mechanics in individuals with medial tibiofemoral osteoarthritis: A proof‐of‐concept study towards personalized interventions
Source: J Orthop Res. 2023 Sep 10;42(2):326–38. doi: 10.1002/jor.25686 (PMC10952410; doi:10.1002/jor.25686)
Supplement: Supplementary file 1 — Supporting Information. [file JOR-42-326-s001.pdf]

Supplementary material for:

Effects of gait modifications on tissue-level knee mechanics in individuals with medial tibiofemoral osteoarthritis: A proof-of-concept study towards personalized interventions

A. Esrafilian*[1], K. S. Halonen[2,3], C. M. Dzialo[3], M. Mannisi[4], M. E. Mononen[1], P. Tanska[1,5], J. Woodburn[6], R. K. Korhonen[1,5], and M. S. Andersen[3,5]

[1]Department of Applied Physics, University of Eastern Finland, Kuopio, Finland
[2]Central hospital of Päijät-Häme, Lahti, Finland
[3]Department of Materials and Production, Aalborg University, Denmark
[4]Medere, Italy
[5]Center for Mathematical Modeling of Knee Osteoarthritis (MathKOA), Aalborg University, Denmark
[6]Griffith Centre of Biomedical and Rehabilitation Engineering, Griffith University, Australia

1. Method

1.1. Material models used within the FE models of the study

A fibril-reinforced poroviscoelastic (FRPVE) material model [1,2] was utilized for the cartilages and the depth-dependent Benninghoff-type (arcade) architecture of collagen fibers was implemented as split-lines for femoral, tibial, and patellar cartilages [3–6]. Menisci were modeled as a fibril-reinforced poroelastic (FRPE) material [1,7]. More details about the implementation of the FRPVE material model can be found from our previous studies [8]. The total stress in the FRP(V)E model (σ_t) consists of the non-fibrillar matrix stress (σ_{nf}), collagen fibril stress (σ_f), and fluid pressure (p):

$$\sigma_t = \sigma_{nf} + \sigma_f - p\mathbf{I} \tag{1}$$

where $\mathbf{I}$ is the unit tensor. The non-fibrillar matrix was modeled by compressible neo-Hookean properties. The stress within the non-fibrillar matrix is given by [2]:

$$\sigma_{nf} = \frac{1}{2}K(J - \frac{1}{J})\mathbf{I} + \frac{G}{J}(\mathbf{F}.\mathbf{F}^T - J^{(2/3)}\mathbf{I}) \tag{2}$$

$$K = \frac{E_{nf}}{3(1 - 2\nu_{nf})} \tag{3}$$

$$G = \frac{E_{nf}}{2(1 + \nu_{nf})} \tag{4}$$

where G and K are the shear and bulk moduli of the non-fibrillar matrix, J is the determinant of the deformation tensor $\mathbf{F}$, E_{nf} and ν_{nf} are Young's modulus and Poisson's ratio of the non-fibrillar matrix, respectively. A strain-dependent permeability (k) [9] is given by:

$$k = k_0(\frac{1 + e}{1 + e_0})^M \tag{5}$$

where k_0 is the initial permeability, e and e_0 are the current and the initial void ratios, and M is a positive constant. The fluid fraction was assumed to be depth-dependent in equilibrium [10] as:

$$n_{(f,eq)} = 0.85 - 0.15d_n \tag{6}$$

where d_n is the normalized depth (0 at the surface and 1 at the cartilage-bone interface).

*amir.esrafilian@uef.fi, esrafilian@gmail.com

The cartilage collagen fibers were modeled as a viscoelastic material. In the material model, a nonlinear spring (with the strain-dependent modulus $E_{\epsilon}\epsilon_f$) is in series with a linear dashpot (with the damping coefficient η). This nonlinear spring-dashpot system is in parallel with a linear spring (with the initial modulus E_0). Fibrils were assumed to only resist tension; thus, the collagen fibril stress of the cartilage was formulated as [2,11]:

$$\sigma_f = \begin{cases} -\frac{\eta}{2\sqrt{(\sigma_f - E_0\epsilon_f)E_{\epsilon}}}\dot{\sigma}_f + E_0\epsilon_f + (\eta + \frac{\eta E_0}{2\sqrt{(\sigma_f - E_0\epsilon_f)E_{\epsilon}}})\dot{\epsilon}_f & , \quad \epsilon_f > 0 \\ 0 & , \quad \epsilon_f \leq 0 \end{cases} \tag{7}$$

where σ_f and ϵ_f are the fibril stress and strain, and $\dot{\sigma}_f$ and $\dot{\epsilon}_f$ are the fibril stress and strain rates.

The collagen fibers within the menisci were modeled as linear elastic (with Young's modulus of E_f). The menisci collagen fiber stress was formulated as [12]:

$$\sigma_f = \begin{cases} E_f\epsilon_f & , \quad \epsilon_f > 0 \\ 0 & , \quad \epsilon_f \leq 0 \end{cases} \tag{8}$$

The collagen fiber network consisted of primary and secondary fibrils [2]. The primary collagen fibrils form a depth-dependent arcade-like structure [13], while the secondary fibrils are randomly organized in 13 different random orientations [2]. Secondary fibrils mainly replicate the inter-fibril connections and cross-links in the collagen network. Consequently, defining C as the amount of the primary fibrils with respect to the secondary fibrils, the stresses are given by [2]:

$$\begin{cases} \sigma_{f,p} = C\sigma_f \\ \sigma_{f,s} = \sigma_f \end{cases} \tag{9}$$

Table S1. Material parameters for the knee joint cartilages and menisci

Material parameter	Femoral cartilage	Tibial cartilage	Patellar cartilage	Menisci
E_{nf}(MPa)	0.215	0.106	0.505	0.5
ν_{nf}(-)	0.15	0.15	0.15	0.36
$k_0(\frac{m^4}{N \cdot s} \times 10^{-15})$	6	18	1.9	1.25
η(MPa · s)	1062	1062	1062	-
E_0(MPa)	0.92	0.18	1.88	-
E_f(MPa)	-	-	-	28
E_{ϵ}(MPa)	150	23.6	597	-
C(-)	12.16	12.16	12.16	12.16
M(-)	5.09	15.64	15.93	5.09
$n_{f,eq}$(-)	$0.85 - 0.15d_n$	$0.85 - 0.15d_n$	$0.85 - 0.15d_n$	0.72

Consistent with our previous study [14], ligaments and tendons were modelled as spring bundles to have sufficient accuracy in the estimated parameters while keeping the computational demand reasonable. Ligament and tendon insertion points were segmented from the template MRIs. Non-linear spring bundles were used to replicate the Anterior cruciate ligament (ACL, 60 springs), posterior cruciate ligament (PCL, 60 springs), lateral collateral ligament (LCL, 18 springs), and medial collateral ligament (MCL, 18 springs). Utilizing a bundle of springs provides the ligament model with compression-tension nonlinearity with different properties along and perpendicular to the fibril/spring directions. The slack, toe, and linear regions of the ligaments were formulated according to the study by Blankevoort et al. [15] as:

$$f_s = \begin{cases} 0 & , \quad \epsilon_s < 0 \\ \frac{1}{4}K_s\epsilon_s^2/\epsilon_l & , \quad 0 \leq \epsilon_s \leq 2\epsilon_l \\ K_s(\epsilon_s - \epsilon_l) & , \quad \epsilon_s \geq 2\epsilon_l \end{cases} \tag{10}$$

where f_s is the tensile force in each ligament element, K_s is the ligament stiffness [15], ϵ_l represents the end of the toe region and was set to 0.03 [16], and ϵ is the current strain in the ligament.

The medial and lateral patellofemoral ligaments (MPFL and LPFL, respectively) were modelled using linear spring bundles with no compressive resistance. The spring stiffness (i.e. as the bundle) was defined as 15.9 N/mm for MPFL and 11.7 N/mm for LPFL [17]. Menisci horn attachments were modelled as linear

spring bundles with a total stiffness of 336 N/mm and 381 N/mm for anterior and posterior sides, respectively [18]. Similarly, the patellar tendon was represented by two springs (no resistance in compression) with a total spring constant equal to 545 N/mm [19].

1.2. Loading, boundary conditions, and finite element analysis

We exploited a kinematics-kinetics driven MS-FE modeling approach, developed and verified in our previous studies [20–22], to provide the FE models with inputs. Inputs to the FE models (Fig. 1) consisted of 1) knee flexion angle, 2) the net forces and moments on femur coming from the gravitational, inertial, muscle, and the hip joint reaction forces, and 3) the net forces and moments applied on patella coming from the gravity, inertia and the quadriceps muscles.

Within the FE models, two reference points (i.e., femoral and patellar) were defined based on the origin of the femoral and patellar coordinate systems of the associated MS models, which were set according to bony landmarks identified from the participants' MRI. The FE models' inputs were applied to the femoral and patellar reference points, correspondingly, while all the nodes on the bottom of the tibia (i.e., either tibial cartilage or tibial subchondral bone) were fixed to the ground (Fig. 1). All the nodes on the inner surfaces of the femoral and patellar subchondral bones (i.e., opposite side of the cartilage-subchondral bone interface) were coupled to the femoral and patellar reference points, respectively (Fig. 1). Also, the nodes at the interface of the cartilage-subchondral bones of the femoral and patellar cartilages were coupled to the femoral and patellar reference points, respectively (Fig. 1). This method of coupling (i.e., instead of tie constraint) substantially reduced convergence issues of the Abaqus solver, as well as computational time (note that we were not interested in bone mechanics). All the couplings were defined using the kinematic coupling of the Abaqus software.

Contact interactions were defined to include all the possible contacts within the FE models, i.e., cartilage-to-cartilage, cartilage-to-menisci, cartilage-to-subchondral bone, and menisci-to-subchondral bone contacts. The femur had 5 DoF, and the patella had 6 DoF in the FE models. While primary knee kinematics (i.e., knee flexion angle) of the FE models was driven using the knee flexion angle estimated by the MS models, secondary knee kinematics were governed by the interaction of knee ligaments with moments and forces applied to the FE models. FE analyses were performed in Abaqus (v 6.20, Dassault Systèmes, US) using soil consolidation analysis, and the whole stance phase of each gait trial was analyzed.

2. Results

Complementary results of the study are illustrated in Figs. S1 to S5.

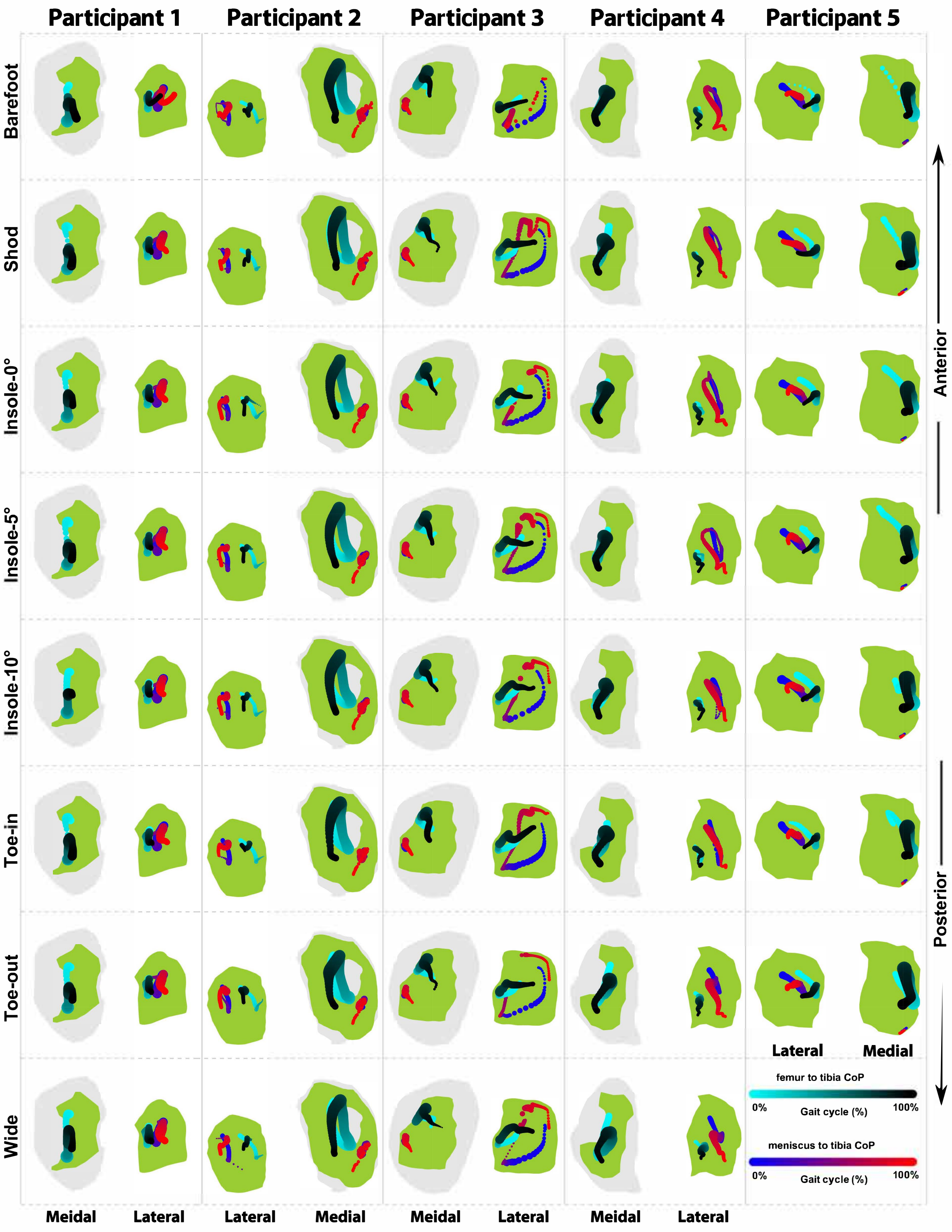

Fig. S1: The center of pressure (CoP) on the tibial cartilage for the patients of the study during walking with different gait modifications. The green shows the tibial cartilage, and the gray shows the subchondral bone. The CoP at the beginning of the cycle is shown in blue for the meniscus to the tibial cartilage contact region and in cyan for the femoral cartilage to the tibial cartilage contact region (that gradually turns red and black, respectively, towards the end of the gait cycle). Note that the thickness of the CoP trace represents the magnitude of the corresponding JCF (e.g., JCF passing through the femoral cartilage to tibial cartilage contact region) at that time point, normalized to the total tibiofemoral JCF. Plots show average profile of each gait modification.

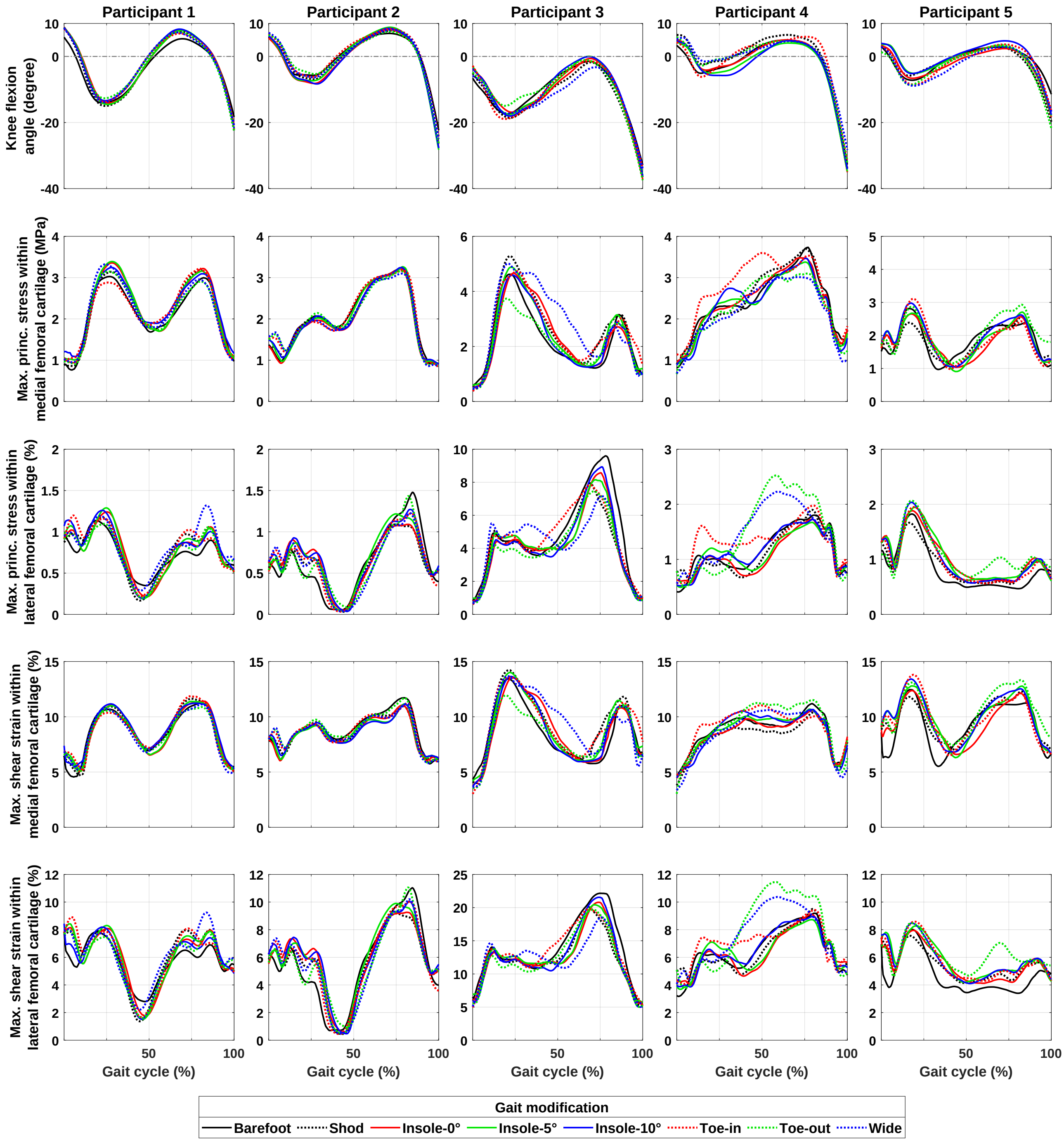

Fig. S2: Knee flexion angle (top row), and the peak of the maximum principal stress (previously suggested as the indicator of collagen network damage) and maximum shear strain (previously suggested as the indicator of proteoglycan loss) within the medial and lateral femoral cartilage of the study participants walking with different gait modifications. Plots show average profile of each gait modification.

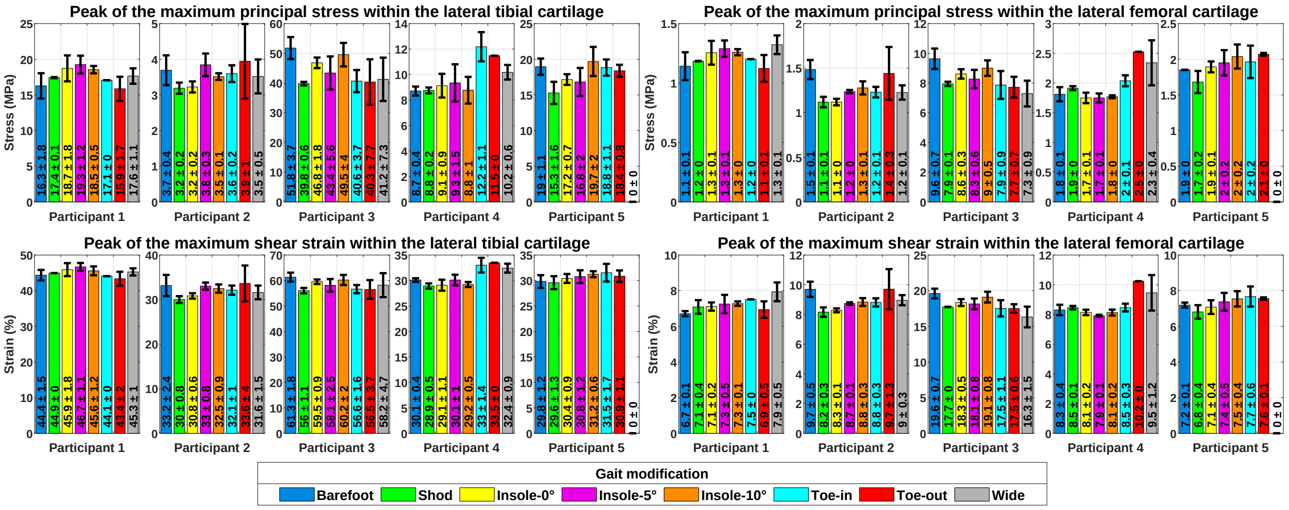

Fig. S3: The peak of the maximum principal stress (top row) and maximum shear strain (bottom row) within the lateral tibial cartilage (on the left) and lateral femoral cartilage (on the right) of study participants during walking with different gait modifications. Error bars show the standard deviation. The corresponding magnitudes (mean ± standard deviation) are shown in the bars for ease of reading.

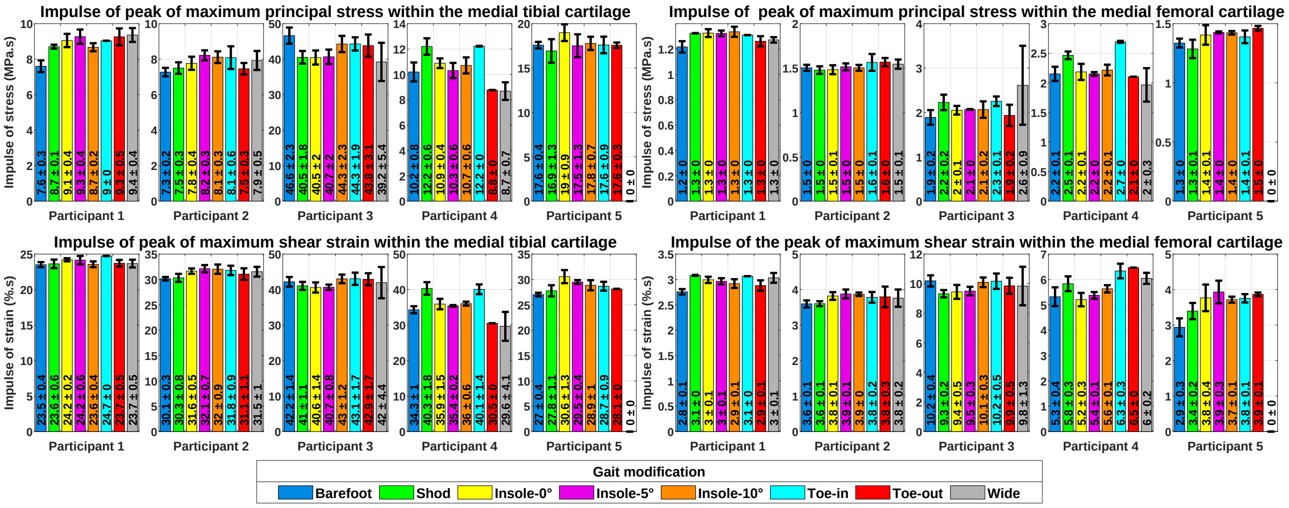

Fig. S4: The impulse of the maximum principal stress (top row) and maximum shear strain (bottom row) within the medial tibial cartilage (on the left) and medial femoral cartilage (on the right) of study participants during walking with different gait modifications. Error bars show the standard deviation. The corresponding magnitudes (mean ± standard deviation) are shown in the bars for ease of reading.

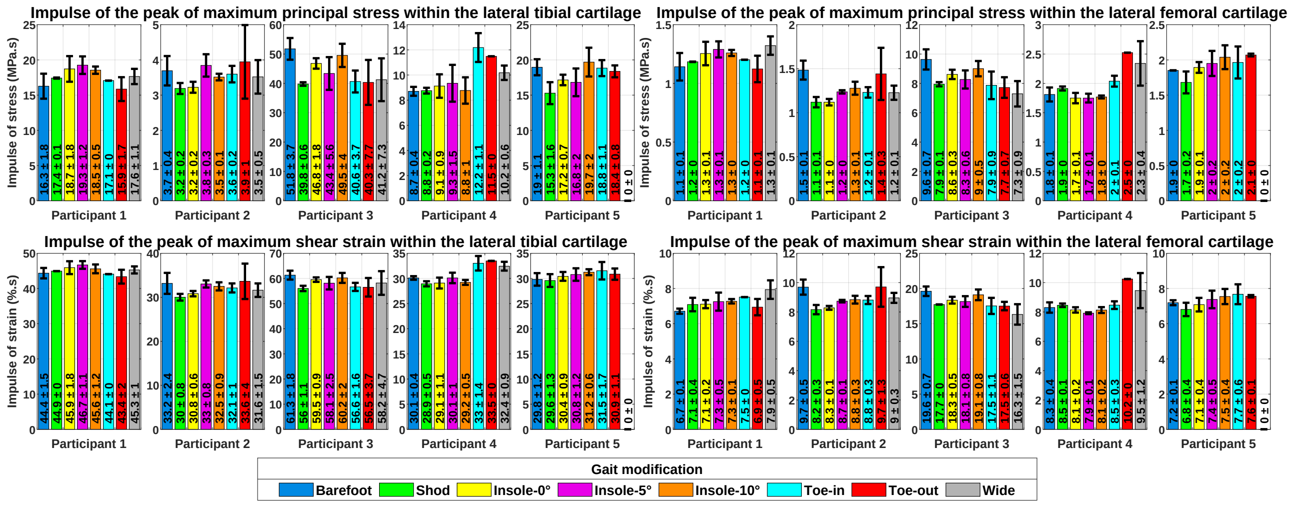

Fig. S5: The impulse of the maximum principal stress (top row) and maximum shear strain (bottom row) within the lateral tibial cartilage (on the left) and lateral femoral cartilage (on the right) of study participants during walking with different gait modifications. Error bars show the standard deviation. The corresponding magnitudes (mean ± standard deviation) are shown in the bars for ease of reading.

References

[1] P. Julkunen, P. Kiviranta, W. Wilson, J. S. Jurvelin, and R. K. Korhonen, "Characterization of articular cartilage by combining microscopic analysis with a fibril-reinforced finite-element model," *Journal of biomechanics*, vol. 40, no. 8, pp. 1862–1870, 2007.

[2] W. Wilson, C. Van Donkelaar, B. Van Rietbergen, K. Ito, and R. Huiskes, "Stresses in the local collagen network of articular cartilage: a poroviscoelastic fibril-reinforced finite element study," *Journal of biomechanics*, vol. 37, no. 3, pp. 357–366, 2004.

[3] S. Below, S. P. Arnoczky, J. Dodds, C. Kooima, and N. Walter, "The split-line pattern of the distal femur: A consideration in the orientation of autologous cartilage grafts," *Arthroscopy: The Journal of Arthroscopic & Related Surgery*, vol. 18, no. 6, pp. 613–617, 2002.

[4] P. Boettcher, M. Zeissler, J. Maierl, V. Grevel, and G. Oechtering, "Mapping of split-line pattern and cartilage thickness of selected donor and recipient sites for autologous osteochondral transplantation in the canine stifle joint," *Veterinary surgery*, vol. 38, no. 6, pp. 696–704, 2009.

[5] D. W. Goodwin, Y. Z. Wadghiri, H. Zhu, C. J. Vinton, E. D. Smith, and J. F. Dunn, "Macroscopic structure of articular cartilage of the tibial plateau: influence of a characteristic matrix architecture on mri appearance," *American Journal of Roentgenology*, vol. 182, no. 2, pp. 311–318, 2004.

[6] B. M. Leo, M. A. Turner, and D. R. Diduch, "Split-line pattern and histologic analysis of a human osteochondral plug graft," *Arthroscopy: The Journal of Arthroscopic & Related Surgery*, vol. 20, pp. 39–45, 2004.

[7] Y. Dabiri and L. Li, "Influences of the depth-dependent material inhomogeneity of articular cartilage on the fluid pressurization in the human knee," *Medical engineering & physics*, vol. 35, no. 11, pp. 1591–1598, 2013.

[8] K. S. Halonen, M. Mononen, J. Jurvelin, J. Töyräs, A. Kłodowski, J.-P. Kulmala, and R. Korhonen, "Importance of patella, quadriceps forces, and depthwise cartilage structure on knee joint motion and cartilage response during gait," *Journal of biomechanical engineering*, vol. 138, no. 7, 2016.

[9] W. Lai, V. C. Mow, and V. Roth, "Effects of nonlinear strain-dependent permeability and rate of compression on the stress behavior of articular cartilage," *Journal of Biomechanical Engineering*, vol. 103, no. 2, pp. 61–66, 1981.

[10] H. Lipshitz, R. Etheredge 3rd, and M. J. Glimcher, "In vitro wear of articular cartilage." *The Journal of bone and joint surgery. American volume*, vol. 57, no. 4, pp. 527–534, 1975.

[11] W. Wilson, C. van Donkelaar, B. van Rietbergen, K. Ito, and R. Huiskes, "Erratum to "stresses in the local collagen network of articular cartilage: a poroviscoelastic fibril-reinforced finite element study" [journal of biomechanics 37 (2004) 357–366] and "a fibril-reinforced poroviscoelastic swelling model for articular cartilage" [journal of biomechanics 38 (2005) 1195–1204]," *Journal of Biomechanics*, vol. 38, no. 10, pp. 2138–2140, 2005.

[12] E. Danso, J. Mäkelä, P. Tanska, M. Mononen, J. Honkanen, J. Jurvelin, J. Töyräs, P. Julkunen, and R. Korhonen, "Characterization of site-specific biomechanical properties of human meniscus—importance of collagen and fluid on mechanical nonlinearities," *Journal of biomechanics*, vol. 48, no. 8, pp. 1499–1507, 2015.

[13] A. Benninghoff, "Form und bau der gelenkknorpel in ihren beziehungen zur funktion," *Zeitschrift für Zellforschung und mikroskopische Anatomie*, vol. 2, no. 5, pp. 783–862, 1925.

[14] A. Esrafilian, L. Stenroth, M. E. Mononen, P. Tanska, S. Van Rossom, D. G. Lloyd, I. Jonkers, and R. K. Korhonen, "12 degrees of freedom muscle force driven fibril-reinforced poroviscoelastic finite element model of the knee joint," *IEEE Transactions on Neural Systems and Rehabilitation Engineering*, vol. 29, pp. 123–133, 2021.

[15] L. Blankevoort and R. Huiskes, "Ligament-bone interaction in a three-dimensional model of the knee," *Journal of biomechanical engineering*, vol. 113, no. 3, pp. 263–269, 1991.

[16] D. L. Butler, M. D. Kay, and D. C. Stouffer, "Comparison of material properties in fascicle-bone units from human patellar tendon and knee ligaments," *Journal of biomechanics*, vol. 19, no. 6, pp. 425–432, 1986.

[17] P. Atkinson, T. Atkinson, C. Huang, and R. Doane, "A comparison of the mechanical and dimensional properties of the human medial and lateral patellofemoral ligaments," in *Proceedings of the 46th Annual Meeting of the Orthopaedic Research Society, Orlando, FL*, 2000.

[18] D. F. Villegas, J. A. Maes, S. D. Magee, and T. L. H. Donahue, "Failure properties and strain distribution analysis of meniscal attachments," *Journal of biomechanics*, vol. 40, no. 12, pp. 2655–2662, 2007.

[19] L. Schatzmann, P. Brunner, and H. Stäubli, "Effect of cyclic preconditioning on the tensile properties of human quadriceps tendons and patellar ligaments," *Knee Surgery, Sports Traumatology, Arthroscopy*, vol. 6, no. 1, pp. S56–S61, 1998.

[20] A. Esrafilian, L. Stenroth, M. E. Mononen, P. E. J. Vartiainen, P. Tanska, P. A. Karjalainen, J.-S. Suomalainen, J. P. A. Arokoski, D. Saxby, D. G. Lloyd, and R. K. Korhonen, "An emg-assisted muscle-force driven finite element analysis pipeline to investigate joint- and tissue-level mechanical responses in functional activities: towards a rapid assessment toolbox," *IEEE Transactions on Biomedical Engineering*, pp. 1–1, 2022.

[21] A. Esrafilian, L. Stenroth, M. E. Mononen, P. Vartiainen, P. Tanska, P. A. Karjalainen, J.-S. Suomalainen, J. P. A. Arokoski, D. J. Saxby, D. G. Lloyd, and R. K. Korhonen, "Toward tailored rehabilitation by implementation of a novel musculoskeletal finite element analysis pipeline," *IEEE Transactions on Neural Systems and Rehabilitation Engineering*, vol. 30, pp. 789–802, 2022.

[22] K. Halonen, C. M. Dzialo, M. Mannisi, M. Venäläinen, M. de Zee, and M. S. Andersen, "Workflow assessing the effect of gait alterations on stresses in the medial tibial cartilage-combined musculoskeletal modelling and finite element analysis," *Scientific reports*, vol. 7, no. 1, pp. 1–14, 2017.
